# Master's level in primary health care education - students' and preceptors' perceptions and experiences of the alteration in the clinical areas

**DOI:** 10.1186/1472-6955-9-11

**Published:** 2010-06-16

**Authors:** Anna Löfmark, Anna-Greta Mamhidir

**Affiliations:** 1 Department of Health and Caring Sciences, University of Gävle, Sweden

## Abstract

**Background:**

Many Western European countries are undergoing reforms with changes in higher education according to the Bologna declaration for Higher European Education Area. In accordance with these changes, the Master's degree was introduced in specialist nurse education in Sweden in 2007, and as a result changed the curriculum and modified theoretical and clinical areas. The aim of this study was to investigate students' and preceptors' perceptions and experiences of Master's level education in primary health care with a focus on the clinical area.

**Methods:**

A descriptive design and qualitative approach was used. Interviews with ten students and ten preceptors were performed twice, before and after the clinical practice period. Interviews were audio-recorded, transcribed verbatim and themes formulated.

**Results:**

Students perceived alteration in the content of the education at the Master's level such as more independence and additional assignments. The preceptors perceived benefits with the Master's level but were unsure of how to transform theoretical and abstract knowledge into practice. Writing the Master's thesis was seen by students to take time away from clinical practice. For some students and preceptors the content of the Master's level clinical practice area was experienced as vague and indistinct. The students had not expected supervision to be different from earlier experiences, while preceptors felt higher demands and requested more knowledge. Both students and preceptors perceived that education at the Master's level might lead to a higher status for the nurses' profession in primary health care.

**Conclusions:**

Students and preceptors experienced both advantages and disadvantages concerning the change in specialist nurse education in primary health care at the Master's level. The altered educational content was experienced as a step forward, but they also questioned how the new knowledge could be used in practice. The relevance of the Master's thesis was questioned. Supervision was seen by students as an introduction to the work of the district nurses' work. Preceptors perceived high demands and did not feel enough qualified for student supervision. Both groups considered it an advantage with the change in education that could result in higher status for nurses working in primary health care.

## Background

Over the last three decades, many Western European countries have implemented extensive reforms in the nursing education system. This is influenced by new challenges and demands in health care such as population ageing, technology growth, increased level of patient knowledge, and evidence based practice in nursing [1]. Other reasons for changes are the current investments within higher education according to the Bologna declaration for Higher European Education Area [2]. The aim is to transform higher education in Europe to be similar where different national systems can share the frameworks for Bachelor's, Master's, and Doctoral levels of education [3].

Until 2007, specialist nurse education in Sweden has been taught as vocational education and training and did not belong in the university system. When the Bologna directives in 2007 were introduced in Sweden, all specialist nurse education consisted of one or one and a half years of study (depending on the professional area) and was transformed and called the Graduate Diploma in Specialist Nursing at the Master's level [4]. This change in specialist nurse education resulted in new demands for teachers in order to incorporate both theoretical and clinical areas.

The clinical area is of special interest, as this is where the students confront professional requirements. For the preceptors, engaged in the students' supervision, it is their arena and they are familiar with what it means to work as a specialist nurse. Therefore, it was of interest to investigate how students and preceptors in a specialist nursing education area perceived and experienced the alteration of educational content when it was merged into the new university system.

Great changes in the nurse education system have occurred as a result of the Bologna declaration signed by the EU Ministries of Education in 1999. It is a framework where different objectives are intended to be met by 2010. The system is organised in cycles, with a common system for credits, called the 'European Credits Transfer System' (ECTS). The credit system is used to describe the students' workload, where 60 ECTS credits can be seen as the workload of an academic year. A model for studies has been proposed among member states based on credits and qualifications: three years for the Bachelor degree consisting of 180 to 240 ECTS credits (first cycle or ground level), and 5 years for the Master's degree consisting of another 60 to 120 ECTS credits (second cycle or advanced level, one or two years of study), and 8 years total for the PhD degree (third cycle) with agreement of at least 300 ETCS credits before entering doctoral level [3].

Inspired by the Bologna challenge, a group of 16 European countries took up the work of identifying generic and specific competencies for the different levels in order to enhance curriculum development. Different disciplines were analysed and nursing was one of them. This project, the Tuning project [3,5], describes competencies to be acquired for the Bachelor's degree and necessary additional competencies for the next levels. The second cycle descriptors focus on development of academic knowledge on an independent basis so as to be able to apply scientific theory and method. A Master graduate will need to have advanced and specialized nursing competencies. Third cycle descriptors point to the need for competencies in research and studies based on empirical work conducted on an independent basis.

The implementation process of the Bologna directives in nursing education has been studied in scientific literature and policy documents [6]. Analysis of the documents from different countries, reveal a striking similarity with regard to difficulties in the implementation processes. Nursing programmes in the first cycle show a rich variation in levels of education, duration of studies and degrees awarded [7]. Advanced degree programmes in nursing (Master's degree and PhD) exist in most Western European countries and are all integrated into universities [7].

Internationally, Master's degree programmes were initiated before the Bologna process started, facilitated by the migration of education into higher education. Traditionally it has been a pathway to a career in research and in the completion of a PhD. However, Drennan [8] found that nurses at Master's level in Ireland saw the Master's degree as part of a their ongoing professional education rather than as a pathway to a PhD. Changes in the delivery of health care and increasing demands from clients have required institutions in different countries to take initiatives in the development of modules within Master level nursing practice courses e.g. in the UK these are called 'Higher Level Practice' [9]. The Canadian perspective is that there is a need to articulate the definition and description of advanced practice nursing [10]. Another name referring to the same phenomenon is 'advanced clinical practice' [11]. Master programmes in nursing have been available for some time, although there are concerns about how it can be characterised and what sort of clinical expertise is evident in advanced practice.

Since 1993 nursing education in Sweden has awarded a Bachelor's degree in Nursing Science. Further education, as for example, specialist nurse education in primary health care or intensive care, has been carried out as vocational training with an emphasis on the practice of the profession. The entry of specialist nursing programmes into the new academic system as a part of the second cycle meant that it was possible to combine a professional degree with a Master's degree (one year). All specialist nurse educations have common objectives described in the Higher Education Ordinance [4]. Students should, for example, demonstrate knowledge of the scientific basis in a special field of interest and insight into current research and development work connected to professional practice. Development work refers to smaller projects concerning, for example, patient care, staff development or evaluation of patient information. This had to be fulfilled in an independent project as a Master's thesis (diploma project). It was also necessary to demonstrate the ability to integrate knowledge and to analyse, assess and deal with complex issues and situations. Self-knowledge was pointed out as important and an ability to be conscious about the need for further knowledge and continuous upgrading of knowledge and learning (life-long learning) (Table 1).

**Table 1:** Objectives for a Graduate Diploma in Specialist Nursing independent of specialisation according to the Higher Education Ordinance [4]

Objectives	Students must demonstrate the knowledge and skills required to work independently as a specialist nurse.
**Knowledge and understanding**	- demonstrate knowledge of the scientific basis of the field and insight into current research and development work, together with knowledge of the connection between science and proven experience and the significance of this connection for professional practice - demonstrate detailed knowledge of planning, leading and coordinating health and medical work
**Skills and abilities**	- demonstrate a deeper ability to identify health care needs and set up care plans, independently and in cooperation with patients and family members - demonstrate an ability to lead and evaluate care measures - demonstrate a deeper ability to initiate, carry out and evaluate health-promoting and preventive work; - demonstrate an ability to integrate knowledge and to analyse, assess and deal with complex issues and situations - demonstrate an ability to assist in and independently perform examinations and treatment, including end-of-life care - demonstrate an ability to teach nursing
**Judgement and approach**	- demonstrate self-knowledge and a capacity for empathy - demonstrate an ability to make intervention assessments based on a holistic approach to the human person and on relevant scientific, social and ethical aspects, paying particular attention to human rights - demonstrate an ability to take a professional approach to patients and their family members - demonstrate an ability to identify their need of further knowledge and to continuously upgrade their capabilities

The preceptor model in clinical practice, which is used in many countries, presupposes at both basic and advanced levels that nurses meet the requirements of academic education. Preceptors are responsible for identifying learning needs and planning learning experiences with the students [12]. Students equipped with knowledge from their theoretical studies need help from experienced preceptors to translate and integrate the theoretical knowledge into the practical arena [13]. The model for supervision presupposes co-operation and when changes are made in the university level system this model for supervision may lead to challenges if preceptors and students have different aims, directions and expectations about supervision.

One of the challenges in clinical supervision is the perception about academic education. Entry of professions with a deeply rooted practical content into the academic setting has often been subject to criticism. The perception that nurses' professional training could quite well be carried out 'at the bedside' is associated with the conception that nurses do not need an academic education like other learned professions such as medicine [14].

Research about the implementation process of the Bologna directives in education in Europe is still limited and the process of implementation of specialist nurse education into the three level university system in Sweden has not to our knowledge been investigated. Nursing in primary health care was chosen for this study as representative of specialist nurse education. The aim of this study was to investigate students' and preceptors' perceptions and experiences of Master's level education in primary health care with a focus on the clinical area.

## Methods

### Design

A descriptive design and qualitative approach was used in order to obtain further insight into the clinical areas of education at an advanced level. This design makes it possible to get extended knowledge and understanding about the meaning people ascribe to actions, beliefs, processes and values in naturalistic settings [15].

### Setting

The participants were students and their preceptors in a county in the middle of Sweden. The students had completed their Bachelor's degree and had at least one year experience as nurses in different areas of health care. The second cycle Master's programme in primary health care education comprises theory and practice areas. The clinical practice period was divided into two periods and included 15 ECTS. The first period was four weeks and the latter six weeks. During clinical practice all students were placed in primary health care centres and all had a preceptor assigned.

### Participants

The inclusion criterion for students was that they were assigned to clinical practice in a primary health care centre. Inclusion criteria for preceptors were that they had received information about the changed level of primary health care education, and had recently worked or currently worked as preceptors. Ten students and ten preceptors were selected as an appropriate number for this type of study. Convenience sampling [16] was used for the recruitment procedure. Before the students' first period of clinical practice all students (n = 16) were verbally informed about the study and asked to participate. The first ten students who volunteered were included. Twelve preceptors met the inclusion criterion and the first ten on the list were asked to participate and all accepted.

Nine of ten students were female, mean age was 38.4+ 7.06 (SD) years. They had been working an average of 7 years as nurses. Five of them had taken other university courses, such as cardiology, anaesthesia and one had a course in supervision.

Nine of ten preceptors were female, mean age was 52.6+8.6 (SD) years. They had worked an average of 28 years as registered nurses with a range from 15 - 38 years and an average of 14 years as district nurses with a range from 10 - 18 years. Two of them had taken a course in supervision.

### Data collection

An interview guide with open ended questions was used for the interviews inspired by Kvale's [17] approach of understanding qualitative research interviews and both groups were asked the same questions, but transformed to suit the 2 groups respectively. The interview guide included demographic characteristics, and focused on the meaning of the change in education. In particular, the participants were encouraged to describe their experiences and perceptions concerning content and demands, values, possibilities and hindrances and if the supervision was influential.

### Procedure

Students and preceptors were interviewed twice, before and after the students' clinical practice periods. The students received written information and were interviewed by a university lecturer, at the university or another agreed upon place. Participating preceptors obtained written information and were interviewed at their recommendation, in the primary health care centres. A nurse manager in primary health care carried out the interviews. The interviewer had a position at an administrative level and was not the preceptors' manager. The students' and preceptors' interviews were tape-recorded, transcribed verbatim and lasted from 30 minutes to 65 minutes.

### Data analysis

Thematic content analysis was used according to the description by Newell and Burnard [15]. At first, each author independently read and re-read student and preceptor transcribed data, in order to be familiar with the text, its ideas and themes. Subsequently, the authors met, shared and discussed mutual themes from the students and the preceptors. According to Newell and Burnard [15] two approaches can be used in the process of thematic analysis. One of the approaches is directed to examine the answers of pre-set questions and the other allows themes to be formulated from the data. In our analysis, the latter approach was adopted which allowed us to grasp similar and different perspectives in text from both the students' and preceptors' perspectives related to our aims. The analysis process was followed (stages 1-6) according to Newell and Burnard [15] (Table 2) and themes formulated reflecting the categorised content. All interviews from each group were treated in the same way as one set of data in the analysis.

**Table 2:** Overview of the thematic content analysis process, stage one to six, guided by Newell and Burnard [15]

**Stage one**	Notes were made after each interview regarding the topics talked about during the interview. This memo was a short note of an idea, theory, thought or feeling that occurred to the researchers. The idea of notes is similar to that of keeping field notes i.e. the memos are useful when writing a report.
**Stage two**	The interview text and notes were read searching for general themes that appear in the transcripts. The aim was in focus and the data was becoming well known. General notes were written at this stage for example. There seem to be discussions about students' and preceptors' attitudes to academic versus clinical practice.
**Stage three**	The researchers read through the interview texts several times and headings were written down to describe different aspects of the content. In this stage the so called 'open coding' was used; words and phrases were written in the margin of the transcripts and summarise or categorise what is being said in the text. Data were categorised i.e. the text was reduced and the researchers made judgements of the reductions.
**Stage four**	A number of categories were overlapping. Some of the categories were so similar that several of the 'open codes' could be placed together under higher order codes. This meant that the codes were reduced. A smaller set of category codes was developed. More than 12 final categories in any project are not ideal since it will be difficult to keep in mind the differences between them.
**Stage five**	In this stage, we returned to the interview text supplied with the shortened list of category codes. Thereafter, the category codes were separated in different sections.
**Stage six**	The organised data formed the material from which this qualitative report is written.

### Rigour

Rigour influences the quality of trustworthiness. Trustworthiness as a criterion should be made intrinsic in a research process, and concepts of credibility, dependability and transferability should be established, according to Polit and Beck [16]. Credibility was ensured through different steps. Both researchers were involved and collaborated in the analyses and interpretation of the data, a form of investigator triangulation, which provides a combination of expertise and diverse research training backgrounds. Representative quotations are also added to illustrate the themes in the results [18].

Dependability refers to stability of data over time [15,16]. Data were collected twice, before and after the clinical practice periods using the same questions. In the analysis of the data, all interviews from respective groups were considered as one set of data. The differences in the answers over time were minor. To expect differences over such a short period of clinical practice is not even expected. Dependability was supported by making the research process transparent and the analysis of data is described in steps (see Table 2). To achieve transferability a thorough description of the context and the participants is needed.

### Ethical considerations

According to the Swedish law, no approval from an Ethics Committee was required for this type of study at the time it was conducted. All participants received written information about the study with the invitation to participate. Participation was voluntary, confidentiality of data was guaranteed and they could withdraw at any time. Before the start of the study the university department management and the primary health care management gave permission for the study.

## Results

Four themes relating to the meaning of Master's level in the clinical practice of primary health care education were formulated based on the interviews among students and preceptors. The themes were: altered content of the education, indistinct content of the Master's level, student supervision, and changed image of nurses' profession in primary health care. Quotations are added to illustrate the themes.

### Altered content of the education

The Master's level in specialist nurse education in primary health care was perceived by some students to mean something new in clinical practice. Students considered it as a step forward, a pressing requirement for increased independence and implications of searching for new knowledge. According to the students, the Master's level also involved added assignments and raised requirements e.g. to review scientific articles related to the assignments. Some students expressed it as beneficial.

'Possibilities to be more critical and questioning' (student 6)

'I have got a new way of thinking' (student 5)

Other students were critical because they could not see the advantage of the innovations and exemplified it by assignments and how they were worked out. They doubted the knowledge of nursing science when it was not perceived to be possible to use it in nursing practice.

'To add scientific articles provides knowledge [of how] to search for them, but does not give a tool to use it in my profession' (student 2)

Some of the preceptors saw benefits from a Master's level. The students' assignments were regarded as an improvement for them as nurses. They mentioned seminars for students concerning evidence based care as especially valuable. Some preceptors stated that nursing theories and evidence based practice were emphasised more than before. They saw that the Master's level could contribute to a useful connection between theory and practice, while others expressed insecurity about how to transform theoretical and abstract knowledge into practice.

'Today they talk so much about nursing theories and evidence based practice and I don't think it is so easy to use this language' (preceptor 3)

'I am not sure that they [the theories] always correspond with practice' (preceptor 5)

The Master's thesis and the writing of it, sometimes called 'writing papers' by the students was emphasised as something new, challenging, which had an influence on clinical practice. Students stated that there were greater requirements since the thesis had to be written and research was involved in their education.

'We are supposed to know how to review the scientific articles' (student 5)

'The Master's thesis is in focus. That is what they [the teachers] want. And they

want to have preceptors with Master's level in primary health care' (student 6)

Students expressed that the academic demands at Master's level requires a thesis and a lot of literature that needs to be read. Some saw the thesis as a benefit while others just saw it as irrelevant. Students thought they developed their critical thinking. On the other hand, they considered that writing the thesis took too much time, time away from clinical practice. Most students mentioned writing the thesis as an obstacle since they could not see the need of the change for themselves or for the district nurses in their work.

'I cannot see any useful advantage for the practical work as a district

nurse' (student 3)

'I won't be a better nurse when I have written a paper' (student 6)

'It would have been better with clinical practice than with a paper' (student 2)

The preceptors saw the Master's thesis both as a good development but also problematic. Some expressed relevance to practice to be of fundamental importance in a Master's level education. Investigations performed by students could lead to quality improvement in primary health care and an increased focus on research issues in education was seen as favourable. A negative aspect, mentioned by all preceptors was the time requirement for writing the thesis.

'The results shown in their Master's thesis can be used in quality improvement in the unit and it is very good if the students could carry out evaluations that we haven't had time to do' (preceptor 1)

'The writing takes so much time that they [students] have not enough time for the patients' (preceptor 7)

'The thesis takes energy and the practical work is set aside' (preceptor 3)

Some preceptors reported that their clinical professional experience had become subordinated at the expense of the academic approach. This was found to be problematic since one of the preceptors' strengths, according to them, is their comprehensive clinical experiences. This subordination was also mirrored by shortened periods of practice.

'I have years of clinical experience but that has little value, now science is in focus' (preceptor 5)

'Too much focus on theory instead of work experience' (preceptor 7)

### Indistinct content for Master's level

For some students the demands of the Master's level in clinical practice were not clear. The content was seen as diffuse, vague and indistinct.

'I don't think there is a great difference, more than that, it is not clearly expressed' (student 1)

'The assignments for this period are not on the Master's level' (student 6)

Some preceptors reported that they could not see any appreciable change so far. They emphasised that the meaning of the change in clinical practice was difficult to grasp and that the curriculum was not always easy to understand.

'It is difficult to see the changes if I compare them with before, I am uncertain what it is' (preceptor 2)

'This is a new education so I am still not used to it and all of us must have time

to understand it, this change is a process and it will take time' (preceptor 1)

'I have a limited insight into what needs to be done according to the curriculum' (preceptor 4)

### Student supervision

Students highlighted the nurses' lengthy experience, all the knowledge they had and wanted more time for reflection together with the preceptors. Supervision for them was to be introduced into the district nurses work. The supervision was not supposed or expected by the students to be different from their earlier experience in the Bachelor nursing education.

'Perhaps I am left more alone, otherwise there has been no difference in the supervision' (student 1)

'Preceptors need Master's level before there will be any noticeable difference in the clinical areas' (preceptor 10)

Some preceptors stated that they were not sure if any changes in clinical supervision were necessary since they already supported the students' assignments and their extended reflective approach.

'I am more involved than before in the students' assignments, I like this talk about critical thinking, it's good for the profession' (preceptor 6)

Other preceptors did not feel they were up to date and requested more knowledge about clinical supervision due to the new demands in clinical practice. They felt uncertain about how to change their own supervision.

'It is necessary for me to take part in a course of clinical supervision so that I can learn and be familiar with the demands' (preceptor 7)

The preceptors saw both obstacles and possibilities related to extended clinical supervision. They felt higher demands and experienced they were faced with conflicting constraints as less time and tight schedules in the situation of clinical supervision. On the other hand, they said, being involved contributed to their own development of knowledge. One of their challenges was how to secure time for supervision due to reduced prerequisites.

'We now help and support the students in their theoretical tasks. There are high

demands and it takes time and we haven't always got the time, we have reduced

the staff, our schedules are so tight but it provides us with new knowledge and

therefore it is also good for us' (preceptor 6)

The preceptors mentioned that they often had been out of educational work for a long time and did not always feel updated regardless of their often comprehensive working experience. They reported that this made them feel unqualified for the job.

'I have an older and another kind of education and we did not talk about nursing

theorists and science and we did not write papers' (preceptor 8)

'I am not sure that I am a sufficient supervisor' (preceptor 7)

### Changed image of nurses' profession in primary health care

The students stated that the change to a Master's level in specialist nurse education in primary health care could influence aspects on the profession as a district nurse and could be seen as a step forward. The profession could gain higher status in the future.

'The profession needs development. It is not possible to stay behind, that will be a vocational training education' (student 4)

The preceptors expressed different views on the importance of status for the profession as a district nurse. Some thought that professional status could increase in the future. Students' assignments and Master's theses could contribute to make the district nurses' subject field more visible and help to develop the profession. Other preceptors were sceptical about the increased emphasis on theoretical content and research. They said that they had not much interest in nursing research since their education in this respect was limited. Theory and research did not correspond with their daily work. They were also unsure if employers were interested in nurses with Master's level education.

'The change in the education broadens district nurses' minds' (preceptor 1)

'The district nurses' work is a practical profession' (preceptor 4)

## Discussion

Specialist nurse education in primary health care at the Master's level was investigated since this was the first of its kind in Sweden. Specialist nurse education had previously been conducted as vocational education and training. How students and preceptors, who were involved in this transformation, experienced this change in the clinical practice area of education was seen to be of importance. Principal findings were that the mandate had benefits according to some of the participants. Students found that the content was new, altered and positive in many ways. What students experienced as 'new' could be influenced by the information they received about the content at the start of the education, as well as their own expectations about the education they had chosen. They felt that how this education was carried out introduced possibilities for new ways of thinking, e.g. critical thinking and questioning. These concepts are highlighted in higher education [19].

Preceptors had a different perspective and could compare the situation with their earlier experiences of supervision. Some of them thought this was a great opportunity to be involved in the students' seminars and assignments. This was a positive experience for them and impressions from seminars discussing evidence based practice may be seen as contributing to new incentives in the development of patient care. There are many examples of opinions in the literature concerning the staff's level of education and preparedness for supervision. The ability in health care to respond to changes, maintain and improve quality is dependent on appropriately trained and supported health care professionals (World Health Organisation (WHO) [20]. Drennan and Hyde [21] indicate that the health service is dependent on highly skilled educated workers with a Master's level who can make an impact on patient care. The authors also found that both university and clinical nursing personnel supported the 'knowledgeable doers' with a high level of theoretical knowledge combined with practical know how [22]. Hardcastle [23] assumed from New Zealand experiences that when more and more registered nurses had Bachelor degrees, further education should aimed at a higher education level. Spencer [24] interviewed nurses, midwifes and health visitors who had participated in a Master of Science programme in professional practice, about their perceptions of the impact of higher education on professional practice. They felt strongly that higher education has a positive effect in practice, but also that time and support was necessary in gaining possible benefit in practice.

Students and preceptors also mentioned that the nurses' profession in primary health care would have new opportunities and higher status depending on the new demands in education. Whyte et al. [25] indicated that a Master's degree can open up job opportunities, professional and personal development. The authors argue that if a Master's programme provides the students with contextually transferable academic and professional skills they are better prepared to meet the demands of contemporary health care. Gerrish and co-authors [26] investigated the role of Master's level of education as a professionalising strategy. A Master's level education indicated credibility, clinical capability and application of influence and leadership, and this was found to strengthen the status of nursing. Interviews with nurse educators teaching at the Master's level also revealed dilemmas that they encountered, such as relevance to practice vs. academic demands and breadth vs. depth on this level [27]. Students in the present study were conscious of future demands. They indicated the strong need for development and that it was not possible to remain as a vocational training education.

The preceptors in our study highlighted that theory and research often did not correspond with their daily work. Comments were also noted that writing a thesis took time at the expense of patient care. Barriers to research utilization have been studied. Kajermo and co-authors [28] described research utilization barriers such as inadequate facilities for implementation, lack of time for reading and implementing findings and experience of lack of authority in the organization. Mulhall [29] discussed the gap between nurse practitioners and researchers and why practitioners remain watchful of the research results. The author maintained that knowledge will continuously be mediated by considerations of utility. As role models, the preceptors' attitudes to research and academic writing may have an influence on the students. The Bologna directives elucidated in the Tuning project are evident in their demands on qualifications for the Master's level. To have knowledge about scientific methods is compulsory at the Master's level [2,5].

The Master's thesis was perceived as a theoretical task that had a far too comprehensive an impact on clinical practice. For many of the students in the present study it was the first contact with academic writing. During the period of the interviews, the students were in the process of preparing and performing their thesis work, which continued throughout the clinical practice periods. They felt it took time away from clinical practice and it was not experienced as a guarantee of better work as a nurse. According to Sachs [30], writing a thesis where the student selects a topic for independent research and demonstrates the degree of topic mastery is a reflection of the student's academic and intellectual maturity. This can be the most challenging task for the students. Writing is seen as one of the most important skills that the questioning and critical nurse can develop [31]. Lundgren and Halvarsson [32] described varying experiences when nursing students were asked about the thesis. However, all students considered that the experience of learning, skills of writing and insight they had gained, was of great value for their coming professional life.

Preceptors saw both benefits and disadvantages with the Master's thesis. Some of them thought that the content could contribute to quality improvement in primary health care, which was appreciated [33,34]. Other preceptors hesitated since the time used for writing could have been used for more practical issues. Writing a thesis was new for both students and preceptors. The majority of nurses had completed post-registration clinical courses but this specialist nurse education within the new academic system was an unknown thing for them. One hallmark of higher education is research-based teaching, which enables students to learn how to question, seek out and evaluate the evidence for practice. This is practised in the Master's thesis.

Important findings in the present study are that the Master's level was experienced as vague and diffuse to some students and preceptors. There were participants in this group of students and preceptors that expressed uncertainties and doubts about the introduction of new content into the clinical area. Some preceptors found the changes difficult to grasp and the curriculum difficult to understand. Information from the university about educational changes is of central importance. Gibbon and Luker [35] discussed fifteen years ago, the first Master's level course for Nurse Clinicians in the UK. They thought that there was a need for objective evaluation of a new course in terms of its contribution to changes in health care. Following these findings, ongoing evaluation of the Master's level education is recommended, including experiences from both students and preceptors.

A situation of frustration and uncertainty in both groups may result in less support for the students, and questioning of the intentions in education. Billay and Myrich [36] presented in a recent integrative review of the preceptor literature significant implications for clinical practice. 'A well developed and thoughtful curriculum is essential for a preceptorship model to succeed in the area of clinical practice. Early and meaningful preceptor involvement, attentive listening, facilitation of learning opportunities, and constructive performance feedback' [[36], p 265] were also approaches essential for student learning.

## Limitations

Convenience sampling in both groups was found appropriate to find variations of the phenomena. However, by using convenience sampling some important aspects of the phenomena may have been uncovered. In a small group the experiences are limited to the group. To assure credibility i.e. how well data and processes of analysis address the intended focus, interviews were chosen for the data collection. The interviews of the students were done by one of the researchers, a senior lecturer familiar with clinical practice, but not involved in the students' education. The preceptors were interviewed by a nurse manager, who had an administrative position in primary health care and not a manager of the preceptors. The interviewers' knowledge of the context facilitated the interview situation to a great extent, but could also be seen as a weakness of the study, since some information could have been taken for granted and not taken into consideration or not noticed. The extent of the data was limited to Swedish Master's level (one year) where the programme is usually two years of study.

## Conclusion

The results from the present study reveal that the students and preceptors in primary health care education had varied perceptions and experiences within the group and also between student and preceptor groups about the change in the curriculum content according to the new university system. In this study of students' and preceptors' views of specialist nurse education in primary health care at Master's level in Sweden, the alteration of the content was seen as a beneficial step forward for students, and required greater involvement of preceptors in the educational process. Writing a Master's thesis, which was a new task in nursing education, was seen to take too much time away from clinical practice. The Master's programme was considered vague in content and difficult to grasp. Student supervision was not expected by the students to be different from earlier experience, while the preceptors saw that it would have greater demands. The changed image of the nurses' profession in primary health care could contribute to higher status for primary health care nurses. The results from this study may to some extent be explained in that it was the first specialist nurse education at the Master's level in Sweden.

Few papers have explicitly discussed Master's level education in a specialist nurse education area on perceptions and experience of students in clinical practice and their preceptors. Findings in the present study may be of use for teachers, employers and students in the planning and evaluation of Master's level education in different specialist nurse education programmes since the new university system has been introduced in many countries in Europe. Further studies and comparisons are warranted and would be of great interest.

## Competing interests

The authors declare that they have no competing interests.

## Authors' contributions

AL and A-GM were responsible for the study conception and design and drafting of the manuscript. AL and A-GM performed the data collection and the data analysis. Both authors read and approved the final manuscript.

## Pre-publication history

The pre-publication history for this paper can be accessed here:

http://www.biomedcentral.com/1472-6955/9/11/prepub
